# Imbalance of antioxidant enzymes activities and trace elements levels in Ghanaian HIV-infected patients

**DOI:** 10.1371/journal.pone.0220181

**Published:** 2019-07-24

**Authors:** Osbourne Quaye, Joshua Agbemefa Kuleape, Evelyn Yayra Bonney, Peter Puplampu, Emmanuel Ayitey Tagoe

**Affiliations:** 1 West African Centre for Cell Biology of Infectious Diseases, Department of Biochemistry, Cell and Molecular Biology, University of Ghana, Legon, Accra, Ghana; 2 Noguchi Memorial Institute for Medical Research, University of Ghana, Accra, Ghana; 3 Department of Medicine, Korle Bu Teaching Hospital, Accra, Ghana; University of Tennessee Health Science Center College of Pharmacy Memphis, UNITED STATES

## Abstract

Human immunodeficiency virus (HIV) infection and antiretroviral therapy (ART) have been associated with high oxidative stress in HIV patients. The disparity in antioxidant-oxidant levels in HIV patients favours viral replication and disease progression. This study aimed at determining the effect of ART on antioxidant enzymes activities and trace elements levels in Ghanaian HIV patients. A total of 242 participants; comprising of 105 HIV-infected patients on ART, 77 HIV-infected ART-naïve, and 60 HIV seronegative controls were recruited for the study. Whole blood was collected and used for haematological profiling, and the determination of CD4^+^ counts, superoxide dismutase (SOD) activity and trace element levels. Serum was used for liver function tests and the determination of glutathione reductase (GR) activity, and plasma was used to estimate reduced glutathione (GSH) levels. Low levels of haemoglobin (HB), hematocrit, mean cell volume (MCV) and mean cell hemoglobin (MCH), and trace elements were found in ART-naïve patients compared to those on ART and the seronegative controls. In the ART-naïve patients, glutathione reductase (GR) activity and reduced glutathione (GSH) level were significantly low compared to patients on ART and seronegative controls. Activity of SOD was significantly reduced in ART-naïve patients compared to those on ART and the control group, and manganese is the only trace element that showed a strong negative correlation with SOD activity and a positive and significant correlation with CD4^+^ count, and therefore needs to be investigated further. The study suggests that assessing antioxidant levels or enzymes activities of patients infected with HIV should be considered during therapy.

## Introduction

Human immunodeficiency virus (HIV) infection and/or highly active antiretroviral therapy (HAART) tilts antioxidant-oxidant balance of the host towards the oxidants, with consequential poor disease outcomes [1,2]. Elevated levels of oxidative stress markers were reported in HIV-infected patients [3,4], and the levels of the markers were associated with low CD4^+^ count and increased viral load [5]. Increased levels of reactive oxygen species (ROS) reported in HIV-infected individuals promoted nucleic acid and lipid oxidations, resulting in elevated levels of 8-Oxoguanine (8-oxoG) and malondialdehyde (MDA), respectively, as evidences [3,6]. The elevated levels of the oxidative stress-induced metabolites have also been shown to independently predict disease progression and mortality in HIV patients [7].

Capacity to clear reactive oxygen species (ROS), either in symptomatic or asymptomatic HIV patients, largely depends on the bioavailability of both endogenous and exogenous antioxidants, as well as effective antioxidant enzymes [3]. The involvement of HIV or its virulent proteins in the perturbation of cellular antioxidant enzymes activities has not been decisive. However, some reports have shown a decrease in activity of the antioxidants enzymes; superoxide dismutase (SOD), catalase (CAT) and glutathione oxidase (GPx) activities have been reported to be lower in HIV-infected individuals [8,9]. Depletion of circulatory endogenous antioxidants, including reduced glutathione (GSH) and vitamins have also been observed in HIV-infected patients, and the lower antioxidant levels correlate with low CD4^+^ counts [9,10]. A recent study from our research group reported that homozygous deletion of GSTM1 and GSTT1 genes has no effect on total GST activity in the serum of HIV-infected individuals, and the observed enzyme activity was attributted to a compensatory mechanism due to other enzymes that play a role in metabolising xenobiotics [11]. Even though it was expected in the previous study that a double deletion of the GST polymorphic gene will result in a decrease in the total GST enzyme activity, and play a role in disease conditions such as cancers [12,13], the contrary finding suggests that antioxidant enzymes activities in HIV patients may be influenced by other factors such as race, geographical location and trace elements.

A number of antioxidant enzymes are metalloenzymes and depend on trace elements for their activities. Trace elements availability is therefore crucial for effective enzymatic activities. The involvement of trace elements has been shown to be key in the activities of super oxide dismutase (SOD) and demonstrated in the three isoforms of the enzyme: a cytosolic copper/zinc form (CuZnSOD), a manganese form (MnSOD) in the mitochondrion, and an extracellular isoform (EC-SOD) which is found in plasma [14].

This current study is based on the premise that antioxidant enzymes activities and trace element levels in Ghanaian HIV patients may differ from what have been reported. We hypothesize that HIV/AIDS disease progression marked by reduced CD4^+^ count will be associated with significantly reduced antioxidant enzymes activities and trace element levels in Ghanaian patients. The study therefore aimed at determining the effect of ART on antioxidant enzymes activities and trace elements levels in Ghanaian HIV patients to establish their relationship with viral replication and/or CD4^+^ count.

## Materials and methods

### Study population and sampling site

Study participants were recruited from the Fevers Unit of the Korle Bu Teaching Hospital, a national referral hospital in Accra, Ghana. The study was approved by the Institutional Review Board (IRB) of the Noguchi Memorial Institute for Medical Research (Study approval number 068/14-5), and all participants signed a consent form after the objectives of the study have been explained to them. A total of 242 participants, comprising of 105 HIV-infected patients on ART, 77 HIV-infected ART-naïve, and 60 HIV seronegative individuals were recruited for the study over a period of six months from September 2015 to February 2016. The sample size calculation was based on a bigger research project which was designed to investigate glutathione S-Transferase (GST) gene polymorphism and antioxidant enzymes activity in HIV/AIDS progression in Ghanaian patients [11], of which this study was part. A prevalence of 30%, a Margin of Error of 6%, and a Confidence Interval Score of 1.96 based on a Confidence level of 95%. The calculation resulted in a sample size of 224 subjects. The study recruited patients who are HIV positive with or without ART but not taking other medications, multivitamins, or mineral supplements for at least 3 months prior to the collection of blood samples. Patients who have been diagnosed with hepatitis/opportunistic infections, and those who consumed alcohol, smoked, had diabetes or kidney problems, had liver dysfunction, and pregnant women were excluded from the study. Blood samples were collected from each recruited individual under aseptic conditions and were divided into ethylenediaminetetraacetic acid (EDTA) and gel separator tubes. The whole blood in EDTA tubes was used for haematological profiling, and CD4^+^ count and trace elements levels determinations. Blood samples in gel separator tubes were kept on ice and allowed to clot, and sera obtained by centrifugation were kept frozen at -20 ^o^C until ready for analyses. The sera were used for antioxidants estimation, antioxidant enzymes activities, as well as liver function tests. Demographic data of the patients were obtained using standard questionnaire, and patients’ treatment regimen was retrieved from medical records after permission has been given by the hospital authority.

### Haematological profile and CD4^+^ Count

Full blood cell count was determined with an ABX Pentra 120 DX automated haematology analyser (Horiba ABX, Montpellier, France), and CD4^+^ count was estimated by a BD FACS machine (BD, Brussels, Belgium).

### Liver function tests

Serum albumin, total protein, alkaline phosphatase (ALP), alanine transaminase (ALT), aspartate transaminase (AST), and gamma glutamyl transferase (GGT) were measured using an automated Biosystems A25 Chemistry Analyser (Madrid, Spain), following the manufacturers’ instructions.

### Super oxide dismutase (SOD)

Super oxide dismutase (SOD) activity was determined using a commercial kit from Sigma-Aldrich (Missouri, USA) following the manufacturer’s protocol. The protocol required that 20 μL of sample solution is added to each sample and a well which is labelled as blank 2, whilst 20 μL of double distilled water was added to two wells that are labelled as blanks 1 and 3. A volume of 200 μL WST Working Solution was added to each well and mixed. Dilution Buffer of 20 μL was added to blanks 2 and 3, and 20 μL of Enzyme Working Solution was added to the samples and blank 1. The reaction mixture was mixed thoroughly and incubated at 37°C for 20 minutes. Absorbance was read at 450 nm using a Varioskan Lux microplate reader (Thermo Scientific, Vantaa, Finland), and SOD activity was calculated as described by the manufacturer. The SOD activity assays were done in duplicates.

### Glutathione reductase (GR) activity

The sample for the glutathione reductase (GR) activity assay was prepared using the method adopted by Buizza et al. [15]. The serum samples were pre-treated to destroy GSH by adding 5 μL of 3% H_2_O_2_ to 100 μL of the sample and incubated at 25 ^o^C for 5 minutes. A volume of 5 μL catalase was added to the reaction mixture and incubated under the same conditions of temperature and incubation time as the pre-treatment. The catalase-treated reaction mixture was used to determine the GR activity using a commercial kit (Abcam, Cambridge, UK). Briefly, 20 μL of the reaction mixture was made up to 50 μL with assay buffer in a 96-well plate, and a 50 μL mix reagent (40 μL GR assay buffer, 2 μL DTNB solution (Ellman’s reagent), 2 μL NADPH-GNERAT solution and 6 μL GSSG solution) was added to each well to a final volume of 100 μL. Absorbance at 405 nm (A1) and time (T1) were taken immediately after the addition of the mix reagent. The reaction was incubation at 25 ^o^C in the dark for 10 minutes (T2) and the absorbance (A2) was taken again. The change in absorbance (ΔA_405nm_) was calculated as A2 –A1. A standard curve for TNB was plotted as described by the protocol, and the ΔA_405nm_ value for each sample was extrapolated from the standard curve to obtain equivalent change in TNB (ΔB). The GR activity was calculated as described by the manufacture.

### Reduced glutathione (GSH) levels

Total Glutathione (GSH+GSSG) was measured using an assay kit (Sigma-Aldrich, Missouri, USA), following the manufacturer’s protocol. Briefly, plasma samples were deproteinized using 5% 5-sulfosalicylic acid, and 10 μL of each sample was added to 150 μL working mixture reagent in a 96 well-plate and incubated at room temperature for 5 minutes. A volume of 50 μL NADPH working solution (0.16 mg/mL) was added to each well and mixed thoroughly, and the plates were read at 405 nm with kinetic read at 1-minute intervals for 5 minutes. A standard curve for GSH was plotted as described by the protocol and ΔA405/min was extrapolated from the curve. The amount of reduced GSH was calculated as described by the manufacturer.

### Estimation of trace elements in whole blood

Trace elements were estimated using atomic absorption spectrometry. Briefly, 2 g of blood samples were digested in a mixture of 6 mL of 65% nitric acid and 1 mL of 30% hydrogen peroxide in polytetraflouroethylene (PTFE) Teflon bombs using milestone microwave labstation ETHOS 900, INSTR: MLS-1200 MEGA. Each of the digested samples was made up to 20 mL with double distilled water and assayed for the presence of Iron (Fe), Manganese (Mn), Selenium (Se) and Copper (Cu) using VARIAN AA 240FS-Atomic Absorption Spectrometer in an acetylene-air flame (FLUKA ANALYTICAL, Sigma-Aldrich Chemie GmbH).

### Statistical analysis

Parametric data were represented as mean ± standard deviation, and One-way ANOVA was used to compare the groups. Pearson’s correlation coefficient was determined between oxidative stress markers, CD4^+^ count, and trace elements. GraphPad Prism version 6 was used for all analyses, and *p*-values < 0.05 were considered as statistically significant.

## Results

### Socio-demographics and patients treatment regimen

Females participants were more than males, whilst the control group (38.7 ± 1.9 years) and ART-naïve patients (41.1 ± 1.4 years) were younger than the patients on ART (45.6 ± 0.9 years) (p ≤0.01). There was however no statistically significant difference between the ages of the control group and the ART-naïve patients (p > 0.05). The demographics of the patients with respect to sex and age have been reported in a previous paper [11]. The average amount of time the subjects have been on ART is approximately 3.7 years.

The antiretroviral drugs that were used by the patients on ART in various combinations included Lamivudine (3TC), Zidovudine (AZT), Efavirenz (EFV), Nevirapine (NVP), Atazanavir (ATV), and Lopinavir (LPV). The combinations were 3TC-EFV, 3TC-NVP, 3TC-AZT-EFV, 3TC-AZT-NVP, 3TC-AZT-ATV, 3TC-AZT-LPV, 3TC-TDF-EFV, 3TC-TDF-EFV-LPV, 3TC-TDF-NVP, 3TC-TDF-NVP-ATZ, 3TC-TDF-LPV and 3TC-LPV.

### Haematological profile and trace elements

Individuals recruited for the study comprised of HIV seropositive patients on ART and ART-naïve, and HIV seronegative controls. ART-naïve patients showed altered haematological indices when compared with patients on ART and the seronegative controls. Low levels of haemoglobin (HB), hematocrit, mean cell volume (MCV) and Mean Corpuscular Hemoglobin (MCH) were observed in the ART-naïve patients compared to the ART and seronegative groups (p < 0.001). Conversely, white blood cells (WBC), red blood cells (RBC), neutrophils and lymphocytes levels were significantly elevated in the ART-naïve patients compared to the patients on ART (p < 0.001) (Table 1). The ART-naïve patients also showed low levels of trace elements (iron, manganese, copper and selenium) than the patients on ART and the seronegative group (p < 0.001) (Table 1). There were increasing levels of the haematological indices and trace elements with increasing CD4^+^ count in HIV seropositive patients (Table 2).

**Table 1:** Haematological and trace elements profiles of HIV patients’ groups and the seronegative individuals.

Parameter	Patients		Control (N = 60)	ANOVA *p*-value
	ART (N = 105)	ART-naïve (N = 77)		
HB (g/dL)	12.70 ± 0.15^ϕ^	10.79 ± 0.25*	12.80 ± 0.23	< 0.001
RBC (10^12^/L)	3.84 ± 0.05^ϕ^*	4.05 ± 0.09*	4.51 ± 0.08	< 0.001
WBC (10^9^/L)	4.82 ± 0.13^ϕ^	5.59 ± 0.40*	3.05 ± 0.49	< 0.001
Hematocrit (%)	37.98 ± 0.48^ϕ^*	32.70 ± 0.70*	42.22 ± 0.66	< 0.001
MCV (f)	100.00 ± 1.21^ϕ^*	81.15 ± 0.96*	94.00 ± 1.13	< 0.001
MCH (pg)	33.36 ± 0.39^ϕ^*	26.17 ± 0.35*	28.46 ± 0.38	< 0.001
Neutrophils (10^9^/L)	1.25 ± 0.08^ϕ^*	1.37 ± 0.14*	0.83 ± 0.06	< 0.001
Lymphocytes (10^9^/L)	3.31 ± 0.11^ϕ^*	3.44 ± 0.19*	1.28 ± 0.08	< 0.001
Iron (μg/dL)	51.12 ± 17.46^ϕ^*	31.05 ± 15.71*	80.57 ± 17.81	< 0.001
Manganese (μg/dL)	63.23 ± 10.84^ϕ^*	25.98 ±11.02*	76.21 ± 10.45	< 0.001
Copper (μg/dL)	35.16 ± 14.28^ϕ^*	23.01 ±14.97*	42.84 ± 13.54	< 0.001
Selenium (μg/dL)	4.32 ± 1.05^ϕ^*	2.88 ± 0.72*	6.20 ± 1.37	< 0.001

*p*-values were obtained from One-way ANOVA by comparing the HIV patient groups with the seronegative controls. Hb: hemoglobin, RBC: red blood cells, WBC: white blood cells, MCV: Mean Cell Volume, MCH: Mean Corpuscular Hemoglobin. Bonferroni multiple comparison was used to compare the three groups.

*mean value compared with seronegative group.

^ϕ^mean value compared between ART and ART-naïve groups. Parameters were presented as mean ± SD.

**Table 2:** Haematological and trace elements profiles of the HIV patients stratified by CD4^+^ counts.

Parameter	CD4^+^ count stratification			ANOVA *p*-value
	< 200 (n = 37)	200–350 (n = 31)	>350 (n = 114)	
HB (g/dL)	10.36 ± 0.46^ϕ^ *	11.96 ± 0.36	12.38 ± 1.54	< 0.001
RBC (10^12^/L)	3.86 ± 0.14^ϕ^ *	3.98 ± 0.13	3.94 ± 0.06	< 0.001
WBC (10^9^/L)	4.60 ± 0.27^ϕ^ *	5.28 ± 0.90	5.28 ± 0.14	< 0.001
Hematocrit (%)	31.51 ± 1.30^ϕ^ *	35.96 ± 0.99*	37.10 ± 0.45	< 0.001
MCV (fL)	82.24 ± 1.37^ϕ^ *	90.50 ± 2.20*	95.62 ± 1.39	< 0.001
MCH (pg)	26.64 ± 0.50^ϕ^ *	29.07 ± 0.93*	31.86 ± 0.46	< 0.001
Neutrophils (10^9^/L)	1.22 ± 0.15^ϕ^ *	1.36 ± 0.26	1.31 ± 0.09	< 0.001
Lymphocytes (10^9^/L)	2.71 ± 0.22^ϕ^*	3.00 ± 0.36*	3.65 ± 0.11	< 0.001
Iron (μg/dL)	29.56 ± 20.13*	38.63 ± 18.30*	53.12 ± 20.74	< 0.001
Manganese (μg/dL)	26.39 ±14.82^ϕ^*	56.19 ± 15.40*	64.01 ±14.23	< 0.001
Copper (μg/dL)	21.24 ± 14.63*	27.95 ± 16.38*	39.07 ± 13.46	< 0.001
Selenium (μg/dL)	2.19 ± 1.17*	3.02 ± 1.83*	4.67 ± 1.81	< 0.001

*p*-values were obtained from One-way ANOVA by comparing the CD4^+^ count stratification. Hb: hemoglobin, RBC: red blood cells, WBC: white blood cells, MCV: Mean Cell Volume, MCH: Mean Cell Hemoglobin. Bonferroni multiple comparison was used to compare the three groups.

*mean value was significantly altered as compared with the group showing CD4^+^ count >350, p < 0.001.

^ϕ^mean value was significantly altered compared with the group showing CD4^+^ count from 200–350. Parameters were presented as mean ± SD. n is the number for the subgroups.

### Liver function tests

The enzymatic activities of ALP, AST and GGT, and the levels of albumin and total protein were significantly elevated in the HIV-positive individuals compared to seronegative controls [*p*-value < 0.001] (Table 3). ALP activity and total protein levels in ART-naïve patients were significantly higher, whereas albumin level was significantly lower in the ART-naïve group compared to those on ART (*p*-value < 0.05). Activities of ALP, AST and GGT in the HIV-positive individuals were decreased with increasing CD4^+^ count, whereas albumin level significantly increased with increasing CD4^+^ count (Table 4).

**Table 3:** Liver function test results of the of HIV patients’ groups and the seronegative individuals.

Parameter	HIV-infected participants		Control (N = 60)	ANOVA *p*-value
	ART (N = 105)	ART naïve (N = 77)		
ALP (U/L)	216.32 ±127.90^ϕ^*	271.11 ± 155.5*	149.01± 67.30	< 0.001
ALT (U/L)	19.93 ±10.01	21.93 ± 11.83	20.82 ± 11.50	0.480
AST (U/L)	39.51 ± 16.93*	42.87 ± 23.15*	27.00 ± 11.20	< 0.001
GGT (U/L)	68.58 ± 33.12*	60.68 ± 31.83*	38.30 ± 14.17	< 0.001
Total protein (g/L)	97.46 ± 14.57^ϕ^*	104.58 ± 13.55*	72.93 ± 16.31	< 0.001
Albumin (g/L)	45.54 ± 8.96^ϕ^*	38.89 ± 6.88	37.91 ± 5.22	< 0.001

*p*-values were obtained from One-way ANOVA by comparing the HIV patient groups with the seronegative controls. ALP = Alkaline phosphatase; ALT = Alanine transaminase; AST = Aspartate transaminase; GGT = Gamma glutamyl transferase. Bonferroni multiple comparison was used to compare the three groups.

*mean value compared with seronegative group.

^ϕ^mean value compared between ART and ART-naïve groups. Parameters were presented as mean ± SD.

**Table 4:** Liver function tests in HIV patients stratified by CD4+ counts.

Parameter	CD4+ count stratification			ANOVA *p*-value
	<200 (n = 37)	200–350 (n = 31)	>350 (n = 114)	
ALP (U/L)	333.21 ± 157.51^ϕ^*	257.60 ± 143.91*	204.14 ±116.93	< 0.001
ALT (U/L)	29.32 ± 13.22^ϕ^*	17.90 ± 9.92	18.80 ± 8.12	< 0.001
AST (U/L)	53.51 ± 36.80*	40.30 ± 27.20	37.01 ± 17.14	0.002
GGT (U/L)	84.40 ± 14.12^ϕ^*	72.50 ± 24.13*	57.13 ± 5.52	< 0.001
Total protein (g/L)	101.61 ± 19.82	101.50 ± 11.53	99.83 ± 14.64	0.764
Albumin (g/L)	36.73 ± 8.54*	40.94 ± 7.41*	45.24 ± 8.21	< 0.001

*p*-values were obtained from One-way ANOVA by comparing the CD4^+^ count stratification. ALP = Alkaline phosphatase; ALT = Alanine transaminase; AST = Aspartate transaminase; GGT = Gamma glutamyl transferase. Bonferroni multiple comparison was used to compare the three groups.

*mean value of the group was significantly altered as compared with other groups, p < 0.001.

^ϕ^mean value was significantly altered compared with the group showing CD4^+^ count from 200–350. Parameters were presented as mean ± SD. n is the number for the subgroups.

### Glutathione reductase (GR) activity

**Fig 1:**
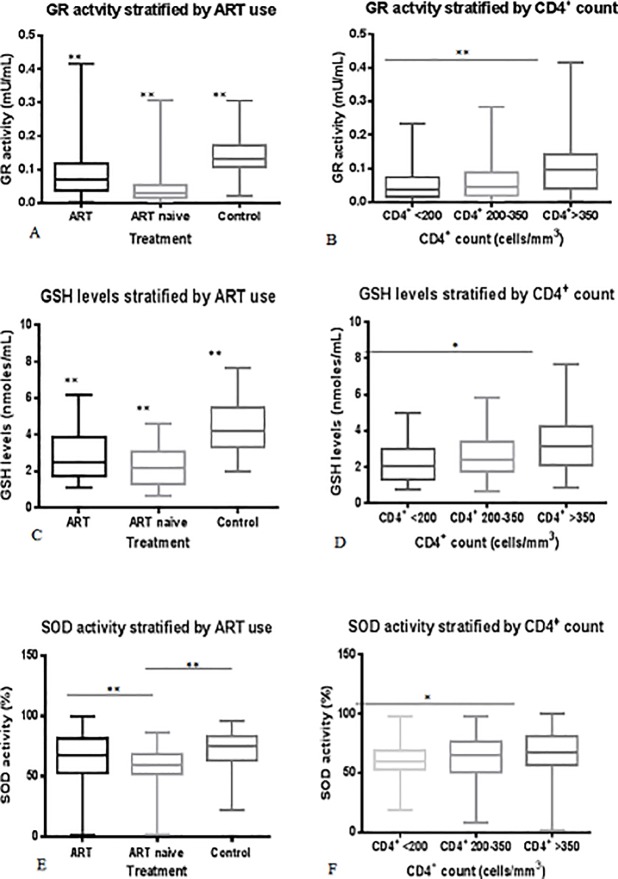
**A-F. Effect of CD4**^**+**^ **count and treatment options on the levels of reduced GSH and antioxidant enzymes activity in HIV-positive patients compared with seronegative group.** Super oxide dismutase (SOD) activity and total glutathione (GSH+GSSG) were determined using commercial kits, and glutathione reductase (GR) activity assay was performed using the method adopted by Buizza et al.

ART-naïve patients showed significantly lower GR activity compared to patients on ART and seronegative controls (p-value < 0.001) (Fig 1A), and the activity increased with increasing CD4^+^ count (*p-*value < 0.001) (Fig 1B, Table 5). The trace elements had a weak correlation with GR activity (Table 5).

**Table 5:** Pearson’s correlation coefficients of CD4^+^ count, antioxidant enzymes activities and trace elements in HIV patients.

Parameters	CD4^+^ count	SOD	GR	GSH	Se	Cu	Fe	Mn
CD4^+^ count	1	0.073	0.187*	0.176*	0.110	0.098	0.045	0.203**
SOD	0.073	1	0.181**	0.308**	-0.013	-0.100	-0.048	-0.241**
GR	0.187*	0.181**	1	0.247**	-0.013	0.030	-0.019	0.009
GSH	0.176*	0.308**	0.247**	1	0.077	-0.003	0.076	-0.039
Se	0.110	-0.013	-0.013	0.077	1	0.242**	0.677**	0.236**
Cu	0.098	-0.100	0.030	-0.003	0.242**	1	0.026	0.492**
Fe	0.045	-0.048	-0.019	0.076	0.677**	0.026	1	-0.012
Mn	0.203**	-0.241**	0.009	-0.039	0.236**	0.492**	-0.012	1

SOD; superoxide dismutase, GR; glutathione reductase, GSH; reduced glutathione, Se: Selenium, Cu; Copper, Fe; Iron, Mn; Manganese. P values

*p<0.05

**p< 0.01

### Reduced glutathione (GSH) level

Reduced glutathione (GSH) level of ART-naive patients was significantly lower than patients on ART and the seronegative controls (p<0.0001) (Fig 1C), and the level increased with increasing CD4^+^ count (p<0.001) (Fig 1D, Table 5). As with the GR activity, the trace elements also had a weak correlation with GSH level (Table 5).

### Superoxide dismutase (SOD) activity

Superoxide dismutase (SOD) activity was significantly reduced in ART-naïve patients compared to those on ART and the control group (*p*-value <0.0001), but there was no significant difference between the SOD levels for the patients on ART and the seronegative control group (p-value > 0.05) (Fig 1E). SOD activity in patients with CD4^+^ count <200 cells/mm^3^ was significantly decreased when compared with patients with CD4^+^ count >350 cells/mm^3^, (p-value < 0.02) (Fig 1F). There was also a weak correlation between the trace elements and SOD, except for manganese which showed a strong negative correlation with SOD activity (*p*-value < 0.01) (Table 5).

## Discussion

Human immunodeficiency virus (HIV) infection has been reported to induce high levels of reactive oxygen species (ROS) in seropositive individuals. The observed disease complications in patients have been attributed to the effects of ROS on tissues [3]. The current study investigated the effect of antiretroviral therapy (ART) on antioxidant enzyme activities and trace elements levels in Ghanaian HIV patients.

Antioxidant enzymes activities are low in ART-naïve HIV-infected patients and high in those on ART. This observation comes from a significant decreased in SOD and GR activities, and GSH levels in the ART-naïve patients compared to patients on ART and seronegative controls. The finding from this study agrees with other studies that have reported reduced antioxidant enzymes activities in HIV-infected patients; ART-naïve individuals showed reduced blood SOD, glutathione oxidase (GPx), catalase (Cat) activities, and elevated malondialdehyde levels compared to healthy controls [4,16]. Increase in antioxidant enzymes activities has been shown to improve the health conditions of HIV positive patients, and antioxidant supplementation was found to decrease lipid peroxidation and improve CD4^+^ counts in ART-naïve HIV-infected patients [17]. Glutathione reductase (GR) is responsible for converting oxidized glutathione (GSSG) to the reduced form (GSH), and therefore low GR activity will lead to elevated GSSG levels, which will result in increased oxidative stress and induce viral replication [5,18]. Administration of ART in HIV management has been shown to induce oxidative stress in patients, however, ART induced oxidative stress is minimal, compared to the level of ROS generated by the viral infection in patients [19–21]. The mechanistic role of oxidative stress in viral replication is through the induction of host transcriptional factor Hypoxia-Inducible Factor-1 (HIF-1) by the HIV protein Vpr to increase mitochondrial activity, resulting in ROS production which activates HIV-1 LTR [22,23].

Manganese seems an important trace element for HIV disease management. The conclusion stems from the fact that the level of manganese was strongly and positively associated with CD4^+^ count. However, the other trace elements, Fe, Se, and Cu, showed a weak correlation with CD4^+^ count, GSH levels, and GR and SOD activities. Almost two decades ago, it was shown that elevated concentrations of manganese inhibited reverse transcription *in vivo* through occupancy of the B site of reverse transcriptase [24]. However, a search through literature did not reveal manganese as a constituent of antiretroviral drugs. Meanwhile the trace element has also been implicated to increase mutation rate in HIV-1 [25]. More work is therefore needed to understand the role of manganese in the improvement of CD4^+^ count in HIV patients.

Stratification analysis revealed higher levels of trace elements in patients with CD4^+^ counts greater than 350 cell/mm^3^, and suggests that the elements are important in improving the health conditions of the patients. In keeping with the results from this study, earlier studies have shown significant reduction in trace elements levels in HIV-infected patients with high viral loads [26–28]. The SOD activity measured in this study included the activities of CuZnSOD and MnSOD, and interestingly a negative correlation between manganese level and total SOD activity was observed. It has been established that HIV Tat protein down regulates the synthesis and induction of MnSOD [29–31]. The decreased SOD activity observed in the HIV patients may be attributed to the inhibition of MnSOD by HIV Tat protein and resulting in the negative correlation between Mn level and SOD activity.

ART-naïve HIV-infected patients were anaemic. This study showed a decreased level of haemoglobin (HB) in ART-naïve patients compared to patients on ART and the seronegative controls. The levels of HB decreased with low CD4+ counts in the HIV-infected individuals and supports previous reports which suggest that anaemia in HIV-infected patients is associated with poor disease outcomes [32–34]. The normal haematological indices observed in patients on ART as compared to the seronegative controls shows a response to treatment and a reduction in oxidative stress-induced red blood cells damage [35,36].

The study showed decreased levels of hemoglobin (HB), hematocrit, mean cell volume (MCV) and mean cell hemoglobin (MCH), with white blood cells (WBC), neutrophils and lymphocytes significantly elevated in the ART-naïve patients than the patients on ART and the control group. The low hematological indices together with the observed low Fe^2+^ levels suggest a microcytic anaemia in the ART-naïve HIV-infected patients and support previous studies that have reported low HB, MCV and MCH in HIV treatment-naïve patients compared to patients on ART [32, 33]. The increased levels of WBC, neutrophils and lymphocytes have been implicated in infection-associated complications [34], and consistent with elevated immune response due to high viral load in the ART-naïve patients. The levels of HB, MCV and MCH decreased with low CD4^+^ counts in the HIV-infected individuals and supports previous reports which suggest that anaemia in HIV-infected patients is associated with poor disease outcomes [35,36]. The low lymphocyte count in patients with low CD4^+^ count confirms that the HIV infection targets T-cells. The normal haematological indices observed in patients on ART as compared to the seronegative controls also suggest a response to treatment and a reduction in red blood cells damage. These observations are in agreement with previous reports [37,38].

The liver plays an important role in detoxification or clearance of ROS through metabolism of compounds which potentially generate ROS and the synthesis of antioxidant enzymes [39]. Liver toxicity was observed to be higher in the ART-naïve HIV-infected patients than those on ART and seronegative controls. The levels of alkaline phosphatase (ALP), aspartate transferase (ASP) and gamma glutamyl transferase (GGT) were significantly elevated in the ART-naïve patients. Even though ART has been implicated in liver damage [40], the increased levels of markers of hepatotoxicity in ART-naïve patients as compared to those on ART could be attributed to the HIV infection exclusively. Advanced stages of HIV/AIDS indicated by reduced levels of CD4^+^ count in patients reported in this study, showed a corresponding elevated markers of hepatotoxicity. HIV-infected patients without any risk factors of liver damage have been shown to have elevated alanine aminotransferase (ALT) level, and this suggests that the infection is the underlying cause of the increased activities of liver enzymes [41,42].

## Conclusion

We observed that antioxidant enzymes activities and trace elements levels were decreased in ART-naïve HIV-infected patients compared to those on ART and increase in manganese level correlates positively with CD4^+^ counts, suggesting that the trace element is important for better disease outcome in HIV patients. The study suggests that ARTs are helpful in the increase of antioxidant enzymes activities in HIV patients. More studies should also be done on manganese to ascertain how the trace element improves CD4^+^ count in HIV patients. Routine monitoring of antioxidant levels or enzymes activities of patients infected with HIV should be done as part of HIV management.

## References

[1] Pace GW, Leaf CD. The role of oxidative stress in HIV disease. *Free Radic Biol Med*. 1995;19(4):523–528. 7590404 10.1016/0891-5849(95)00047-2

[2] Dröge W, Eck H-P, Mihm S. Oxidant-antioxidant status in human immunodeficiency virus infection. *Methods in Enzymol*. 1994;233:594–601. 7912408 10.1016/s0076-6879(94)33062-x

[3] Ivanov AV, Valuev-Elliston VT, Ivanova ON, Kochetkov SN, Starodubova ES, Bartosch B, et al Oxidative stress during HIV infection: mechanisms and consequences. *Oxid Med Cell Longev*. 2016;2:1–18 10.1155/2016/8910396 PMC5088339 27829986

[4] Awodele O, Olayemi SO, Nwite JA, Adeyemo TA. Investigation of the levels of oxidative stress parameters in HIV and HIV-TB co-infected patients. *J Infect Dev Ctries*. 2012; 6(1):79–85. 22240433 10.3855/jidc.1906

[5] Rajopadhye S, Mukherjee S, Chowdhary A. Oxidative stress in HIV/AIDS patients in Mumbai, India. *J Immuno Virol*. 2015; 1(1): 555553–555559.

[6] Watanabe LM., Barbosa JF, Jordão AA., Navarro AM. Influence of HIV infection and the use of antiretroviral therapy on selenium and selenomethionine concentrations and antioxidant protection. *Nutrition*. 2016; 32(11–12):1238–1242. 10.1016/j.nut.2016.03.024 27255831

[7] Masiá M, Padilla S, Fernández M, Rodríguez C, Moreno A, Oteo JA, et al Oxidative stress predicts all-cause mortality in HIV-infected patients. *PLoS ONE*. 2016;11(4):1–12. 10.1371/journal.pone.0153456 PMC4844170 27111769

[8] Baum MK, Sales S, Jayaweera DT, Lai S, Bradwin G, Rafie C, et al Coinfection with hepatitis C virus (HCV), oxidative stress and antioxidant status in HIV-positive drug users in Miami. *HIV Med*. 2012;12(2):78–86. 10.1111/j.1468-1293.2010.00849.x PMC2974022 20500231

[9] Gwarzo MY, Muhammad SA. Extracellular superoxide dismutase activity and plasma malondialdehyde in human immunodeficiency virus subjects of Kano State as surrogate markers of CD4 status. *Clin Dev Immunol*. 2010;6(4):294–300. PMC3615290 23675205

[10] Morris D, Guerra C, Donohue C, Oh H, Khurasany M, Venketaraman V. Unveiling the mechanisms for decreased glutathione in individuals with HIV infection. *Clin Dev Immunol*. 2012;2012:1–10. 10.1155/2012/734125 PMC3254057 22242038

[11] Kuleape JA., Tagoe EA, Puplampu P, Bonney EY, Quaye O. Homozygous deletion of both GSTM1 and GSTT1 genes is associated with higher CD4^+^ T cell counts in Ghanaian HIV patients. *PLoS ONE*. 2018;13(5):1–10. 10.1371/journal.pone.0195954 PMC5967833 29795558

[12] Soto-Quintana O, ZuÂ ñiga-GonzaÂlez G, RamõÂrez-Patiño R, Ramos-Silva A, Figuera LE, Carrillo-Moreno DI, et al Association of the GSTM1 null polymorphism with breast cancer in a Mexican population. *Genet Mol Res*. 2015; 14(4):13066–75. 10.4238/2015.October.26.2 26535619

[13] Khabaz M, Nedjadi T, Gari M, Al-Maghrabi JA, Atta HM, Bakarman M, et al GSTM1 gene polymorphism and the risk of colorectal cancer in a Saudi Arabian population. *Genet Mol Res*. 2016; 15:1. 10.4238/gmr.15017551 26909940

[14] Hassan HM. Biosynthesis and regulation of superoxide dismutases. *Free Rad Biol Med*. 1988;5(5–6):377–385. 2855738 10.1016/0891-5849(88)90111-6

[15] Buizza L, Cenini G, Lanni C, Ferrari-Toninelli G, Prandelli C, Govoni S, et al Conformational altered p53 as an early marker of oxidative stress in Alzheimer's disease. *PLoS ONE*. 2012;7(1):e29789 10.1371/journal.pone.0029789 22242180 PMC3252333

[16] Kostyushov VV., Bokal II, Petrov SA. The study of activity of blood antioxidant enzymes in HIV infection. *Biochemistry (Moscow)*. 2011;5(2):193–196.

[17] Nkengfack G, Ngogang J, Englert H. Effects of 5 a day fruit and vegetable intake on micronutrient level and oxidative stress markers in HIV positive patients: a cluster randomized trial. *Oxid Antioxid Med Sci*. 2013;2(4):275.

[18] Brady TC, Chang LY, Day BJ, Crapo JD. Extracellular superoxide dismutase is upregulated with inducible nitric oxide synthase after NF-kappa B activation. *Am J Physiol*. 1997;273(5 Pt 1):L1002–1006. 10.1152/ajplung.1997.273.5.L1002 9374727

[19] Popoola TD, Awodele O. Interplay between antiretroviral therapy and oxidative stress in HIV seropositive patients. *Afr J Med Med Sci*. 2016;45(1):5–21. 28686824

[20] Sharma B. Oxidative stress in HIV patients receiving antiretroviral therapy. *Curr HIV Res*. 2014;12(1):13–21. 24694264 10.2174/1570162x12666140402100959

[21] Ngondi JL, Oben J, Forkah DM, Etame LH, Mbanya D. The effect of different combination therapies on oxidative stress markers in HIV infected patients in Cameroon. *AIDS Res Ther*. 2006;3(1):1–7. 16859567 10.1186/1742-6405-3-19 PMC1557529

[22] Kurata S. Sensitization of the HIV-1 LTR upon long term low dose oxidative stress. *J Biol Chem*. 1996;271(36):21798–21801. 10.1074/jbc.271.36.21798 8702977

[23] Qutub AA, Popel AS. Reactive oxygen species regulate hypoxia-inducible factor 1 differentially in cancer and ischemia. *Mol Cell Biol*. 2008;28(16):5106–5119. 10.1128/MCB.00060-08 18559422 PMC2519710

[24] Bolton EC, Mildvan AS, Boeke JD. Inhibition of reverse transcription in vivo by elevated manganese ion concentration. *Mol Cell*. 2002;9(4):879–89. 11983178 10.1016/s1097-2765(02)00495-1

[25] Vartanian JP, Sala M, Henry M, Wain-Hobson S, Meyerhans A. Manganese cations increase the mutation rate of human immunodeficiency virus type 1 ex vivo. *J Gen Virol*. 1999;80 (Pt 8):1983–6. 10466794 10.1099/0022-1317-80-8-1983

[26] Saad K., Hammad E, Hassan AF, Badry R. Trace element, oxidant, and antioxidant enzyme values in blood of children with refractory epilepsy. *Int J Neurosci*. 2014;124(3):181–186. 10.3109/00207454.2013.831851 23919524

[27] Olaniyi JA, Arinola OG. Essential trace elements and antioxidant status in relation to severity of HIV in Nigerian patients. *Med Princ Pract*. 2007; 16(6):420–425. 10.1159/000107745 17917440

[28] Han SG, Kim Y, Kashon ML, Pack DL, Castranova V, Vallyathan V. Correlates of Oxidative stress and free-radical activity in serum from asymptomatic shipyard welders. *Am J Respir Crit Care Med*. 2005;172(24):1541–1548. 16166614 10.1164/rccm.200409-1222OC

[29] Flores SC, Marecki JC, Harper KP, Bose SK, Nelson SK, McCord JM. Tat protein of human immunodeficiency virus type 1 represses expression of manganese superoxide dismutase in HeLa cells. *Proc Natl Acad Sci*. 1993;90(16):7632–7636. 10.1073/pnas.90.16.7632 8395050 PMC47196

[30] Marklund S. Regulation by cytokines of extracellular superoxide dismutase and other superoxide dismutase isoenzymes in fibroblasts. *J Biol Chem*. 1992;267(10):6696–6701. 1551878

[31] Brady TC, Chang L-Y, Day BJ, Crapo JD. Extracellular superoxide dismutase is upregulated with inducible nitric oxide synthase after NF-κB activation. *Am J Physiol Lung Cell Mol Physiol*. 1997;273(5):L1002–L1006. 10.1152/ajplung.1997.273.5.L1002 9374727

[32] Enawgaw B, Alem M, Addis Z, Melku M. Determination of hematological and immunological parameters among HIV positive patients taking highly active antiretroviral treatment and treatment naïve in the antiretroviral therapy clinic of Gondar University Hospital, Gondar, Northwest Ethiopia: A comparative cross-sectional study. *BMC Hematol*. 2014;14(1):8 10.1186/2052-1839-14-8 24666771 PMC3994311

[33] Johannessen A, Naman E, Gundersen SG, Bruun JN. Antiretroviral treatment reverses HIV-associated anemia in rural Tanzania. *BMC Infect Dis*. 2011;11(1):190. 21745396 10.1186/1471-2334-11-190 PMC3145581

[34] Tobón-Castaño A, Mesa-Echeverry E and Miranda-Arboleda AF. Leukogram Profile and Clinical Status in vivax and falciparum Malaria Patients from Colombia. *J Trop Med*. 2015;2015(i):18–20. 10.1155/2015/796182 PMC4667023 26664413

[35] Obirikorang C, Yeboah FA. Blood haemoglobin measurement as a predictive indicator for the progression of HIV/AIDS in resource-limited setting. *J Biomed Sci*. 2009;16(1):102. 19922646 10.1186/1423-0127-16-102 PMC2783029

[36] Kreuzer K-A, Rockstroh J. Pathogenesis and pathophysiology of anemia in HIV infection. *Ann Hematol*. 1997;75(5):179–87. 9433373 10.1007/s002770050340

[37] Forna F, Moore D, Mermin J, Brooks JT, Were W, Buchacz K, et al Hematologic changes associated with Zidovudine following single-drug substitution from stavudine in a home-based AIDS care program in rural Uganda. *J Int Assoc Physicians AIDS Care*. 2009;8(2):128–38. 10.1177/1545109709333081 19270152

[38] Redig AJ, Berliner N. Pathogenesis and clinical implications of HIV-related anemia in 2013. *Am Soc Hematol Educ Program*. 2013;2013:377–81. 10.1182/asheducation-2013.1.377 24319207

[39] Muriel P. Role of free radicals in liver diseases. Hepatol Int. 2009; 3(4):526–536. 10.1007/s12072-009-9158-6 19941170 PMC2790593

[40] Mataranyika PA, Kibuule D, Kalemeera F, Kaurac H. Godman B, Rennie TW. Liver enzyme elevations in cohort of HIV/AIDS patients on first-line antiretroviral therapy in Namibia: Findings and implications. *Alexandria J Med*. 2017;54(1):49–56.

[41] Cichoz-Lach H, Michalak A. (2014). Oxidative stress as a crucial factor in liver diseases. World J Gastroenterol. 2014;20(25):8082–8091. 10.3748/wjg.v20.i25.8082 25009380 PMC4081679

[42] Nagu TJ, Kanyangarara M, Hawkins C, Hertmark E, Chalamila G, Spiegelman D, et al Elevated alanine aminotransferase in antiretroviral-naïve HIV infected African patients: magnitude and risk factors. *HIV Med*. 2012;13(9):541–548. 10.1111/j.1468-1293.2012.01006.x 22416813 PMC3391335

